# MicroRNA expression changes in the development of rotator cuff tendon injuries

**DOI:** 10.1016/j.xrrt.2023.03.006

**Published:** 2023-04-23

**Authors:** Giuseppe Francesco Papalia, Edoardo Franceschetti, Giancarlo Giurazza, Francesco Rosario Parisi, Pietro Gregori, Biagio Zampogna, Umile Giuseppe Longo, Rocco Papalia

**Affiliations:** a Department of Medicine and Surgery, Università Campus Bio-Medico di Roma, Roma, Italy; b Research Unit of Orthopaedic and Trauma Surgery, Department of Medicine and Surgery, Università Campus Bio-Medico di Roma, Roma, Italy

**Keywords:** MicroRNA, Biomarker, Expression changes, Rotator cuff, Tendinopathy, Tendon injuries

## Abstract

Traumatic or degenerative rotator cuff (RC) tendon injuries are a leading cause of persistent shoulder pain and reduction of mobility with associated disability and dysfunction, which require each year more than 250,000 surgical repairs in the United States. MicroRNAs (miRNAs) are small noncoding RNAs, that in the posttranscriptional phase lead to the development and function of tissues. The aim of this review was to identify miRNA expression changes in patients with RC pathologies and to determine their relevance as a potential novel diagnostic and potentially therapeutic tool for RC disorders. Various miRNAs seemed to be key regulators in the muscle architecture, determining several modifications in muscle atrophy, skeletal muscle mechanical adaptation, lipid accumulation, and fibrosis in the presence of RC tears. The search was executed using PubMed, Medline, Scopus, and Cochrane Central. We included studies written in English that evaluated the role of miRNA in diagnosis, physiopathology, and potential therapeutic application of RC tendon injuries. We included 11 studies in this review. Many miRNAs emerged as key regulators in the pathogenesis of RC tears, inflammation, and muscle fatty degeneration. In fact, they are involved in the regulation of myogenesis, inflammatory cytokines, metalloproteases expression, muscle adaptation, adipogenesis, fibrogenic factors, and extracellular matrix synthesis. The gene expression may be altered in the pathological processes of tendon lesions. Therefore, the knowledge of all the gene mechanisms underlying RC tendinopathy should be achieved with future diagnostic and clinical studies.

Traumatic or degenerative rotator cuff tendon injuries (RCTI) are a leading cause of persistent shoulder pain and reduction of mobility with associated disability and dysfunction. These tears represent a serious health problem and a significant economic burden. Due to the prevalence of these lesions, each year more than 250,000 rotator cuff (RC) repairs are performed in the United States.^17^^,^^24^ Previous studies have examined the molecular mutations interested in tendon lesions and nonhealing cuff tears.^6^^,^^8^^,^^23^^,^^36^ A better comprehension of gene regulation in normal and injured tendons should be a new frontier for the development of diagnostic tools and therapeutic approaches in the treatment of RCTI. In clinical and experimental models, injuries of tendon fibers have shown alterations in the expression of COL1/3, metalloproteinases (MMPs), and tissue inhibitors of metalloproteinases, due to inflammatory responses and modification of the extracellular matrix (ECM) composition.^1^^,^^30^ Fatty degeneration has been described as a possible risk factor for favorable outcomes of surgical interventions for rotator cuff tears (RCTs).^13^ Goutallier et al^9^ showed a direct relationship between the degree of fatty infiltration and the risk of unsatisfactory outcomes. Therefore, in light of this, the novel therapeutic strategies for RCTI should involve approaches for the prevention or reduction of fatty infiltration.

MicroRNAs (miRNAs) are small noncoding RNAs, that in the posttranscriptional phase link to 3 'untranslated region of a protein-coding mRNA, leading to the development and function of tissues.^5^ Various miRNAs seemed to be key regulators in the muscle architecture, determining several modifications in muscle atrophy, skeletal muscle mechanical adaptation,^19^ lipid accumulation,^20^ and fibrosis^4^ in the presence of RCTs. Therefore, the aim of this review was to identify miRNA expression changes in patients with RC pathologies and determine their relevance as a potential novel diagnostic and potentially therapeutic tool for RC disorders.

## Materials and methods

This review was performed following the Preferred Reporting Items for Systematic Reviews and Meta-Analysis guidelines. The search was executed on 31 July 2022 using PubMed, Medline, Scopus, and Cochrane Central by two independent reviewers (G.F.P. and G.G.). The search strings used were: ("micrornas" [MeSH Terms] OR "micrornas" [All Fields] OR "mirna" [All Fields] OR "mirnas" [All Fields] OR "mirna s" [All Fields]) AND ("rotator cuff" [MeSH Terms] OR ("rotator" [All Fields] AND "cuff" [All Fields]) OR "rotator cuff" [All Fields]). We included studies written in English that evaluated the role of miRNA in diagnosis, physiopathology and potential therapeutic application of RCTI. We excluded meta-analysis, reviews, case reports, and technical notes. The following data were collected: type of miRNA, their function, expression in rotator cuff tear, and effect on rotator cuff pathology.

## Results

**Figure 1:**
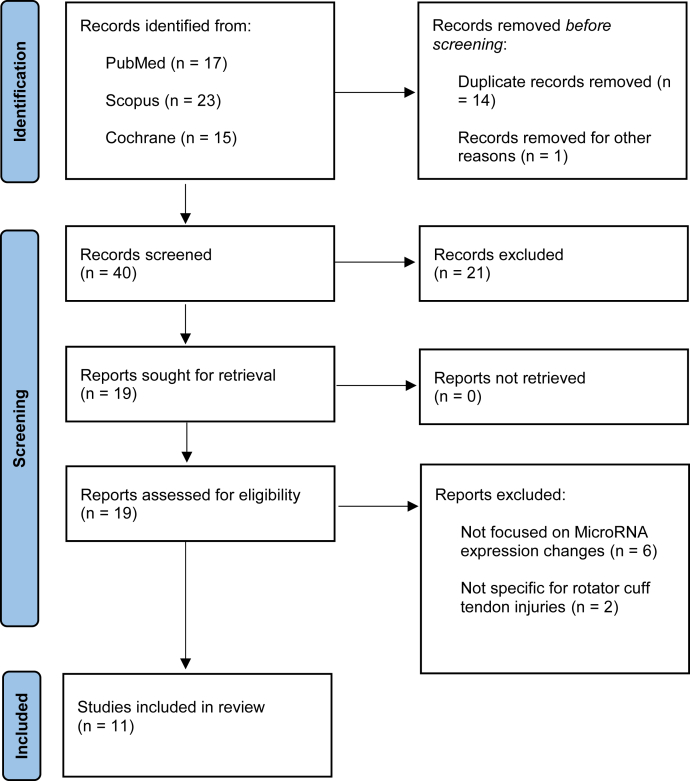
PRISMA 2020 flow diagram. *PRISMA*, preferred reporting items for systematic reviews and meta-analysis.

The literature search identified 55 articles. After duplicates were removed, 40 papers were screened for title and abstract. At this point, we excluded 4 reviews, 3 studies focused on exosomes expression, and 14 studies that were not appropriate for the study design. Therefore, 19 eligible manuscripts were read in full text. Then six studies not focused on microRNA expression changes and two studies not specific to rotator cuff pathology were excluded. Finally, 11 studies were included in this review (Fig. 1). Many miRNAs emerged as key regulators in the pathogenesis of rotator cuff tears, inflammation, and muscle fatty degeneration (Table I).

**Table I:** The effects of miRNAs on rotator cuff tendon pathology.

microRNA	Function	Expression	Effect on rotator cuff pathology	Reference
miR-15a-5p	Regulation of AMPK and TREM-1 in tendon tissue	Downregulated in RC injury with fatty infiltration	Fatty infiltration and inflammation in RC tendon injury	Genes interconnecting AMPK and TREM-1 and associated microRNAs in rotator cuff tendon injury
miR-16-5p				
miR-31-5p				
miR-99a-5p				
miR-100-5p				
miR-103a-3p				
miR-150-5p				
miR-193b-3p				
miR-195-5p				
miR-497-5p				
Let-7b-5p	Regulation of AMPK and TREM-1 in tendon tissue, regulation of myogenesis	Downregulated in RC injury with fatty infiltration	Fatty infiltration and inflammation in RC tendon injuries	Genes interconnecting AMPK and TREM-1 and associated microRNAs in rotator cuff tendon injury
Let-7	Regulation of myogenesis	Upregulated in torn RC muscles	Muscle atrophy and reduction in muscle fiber force production	Aging-associated exacerbation in fatty degeneration and infiltration following rotator cuff tear
miR-18b-5p	Negatively correlates with age and expression of profibrotic genes	Downregulated only in degenerative RC tear and not in RC chronic tendinopathy	Overexpression of profibrotic genes	MicroRNA profiling reveals distinct signature in degenerative rotator cuff pathologies
miR-19a-3p	Negative regulation of JAK-STAT signaling pathway and inflammation related cytokines release (eg, IL-6 or MMP-3)	Downregulated only in degenerative RC tear and not in rotator cuff chronic tendinopathy	Increased expression of inflammatory cytokines	MicroRNA profiling reveals distinct signature in degenerative rotator cuff pathologies
miR-19b-3p				
miR-21	Promotion of ECM synthesis	Upregulated in torn RC muscles	Fibrosis	Aging-associated exacerbation in fatty degeneration and infiltration following rotator cuff tear
miR-31				
miR-214				
miR-381				
miR-23b-3p	Regulatory role in inflammatory response by targeting key inflammatory genes JAK2/STAT3 and interconnecting pathways	Downregulated in glenohumeral arthritis due to massive RC tears	Grater severity of inflammation in glenohumeral arthritis tendon	MicroRNAs associated with inflammation in shoulder tendinopathy and glenohumeral arthritis
miR-100-5p				
miR-145-5p				
miR-146a-5p				
miR 150-5p				
miR 181a-5p				
miR 193b-3p				
Let-7d-5p				
miR-498	Regulatory role in inflammatory response by targeting key inflammatory genes JAK2/STAT3 and interconnecting pathways	Upregulated in glenohumeral arthritis due to massive RC tears	Grater severity of inflammation in glenohumeral arthritis tendon	MicroRNAs associated with inflammation in shoulder tendinopathy and glenohumeral arthritis
miR-297				
miR-25-3p	Downregulation of inflammatory cytokines (eg, TNF-alfa or high mobility group protein HMGB)	Downregulated only in degenerative RC tear and not in rotator cuff chronic tendinopathy	Increased expression of inflammatory cytokines and HMGB1	MicroRNA profiling reveals distinct signature in degenerative rotator cuff pathologies
miR-29a-3p	Regulation of collagen 3 (Col-3) expression in tenocytes, by controlling IL-33 synthesis	Downregulated only in degenerative RC tear and not in RC chronic tendinopathy	Overexpression of Col-3, resulting in an unbalanced Col-1/Col-3 ratio, which is a hallmark of tendon degeneration	MicroRNA profiling reveals distinct signature in degenerative rotator cuff pathologies
miR-29c-3p				
miR-29a	Downregulation of fibrogenic factors, COL-3 expression, vessel formation and ECM production	Downregulated in RC tears with stiffness and not downregulated in RC tears without stiffness	Subacromial bursa inflammation and fibrosis in RC tears with shoulder stiffness	MicroRNA-29a mitigates subacromial bursa fibrosis in rotator cuff lesion with shoulder stiffness
miR-30a-5p	Promotion of cancer cell apoptosis and inhibition of cell proliferation and fibrogenesis	Downregulated in degenerative RC tear and in RC chronic tendinopathy (progressive decline in the expression levels depending on the severity of tendon degeneration)	Extensive and aberrant ECM deposition resulting in fibrosis	MicroRNA profiling reveals distinct signature in degenerative rotator cuff pathologies
miR-93-5p	Promotion of cell survival and proliferation and downregulation of inflammatory response by controlling the expression of Interleukin 1 receptor associated kinase 4 (IRAK4)	Downregulated only in degenerative RC tear and not in RC chronic tendinopathy	Hypocellularity, higher rates of tenocyte apoptosis, premature cellular senescence and inflammation	MicroRNA profiling reveals distinct signature in degenerative rotator cuff pathologies
miR-130a	Suppression of adipogenesis by inhibiting the translation of PPAR-g mRNA in adipocytes	Upregulated in torn rotator cuff muscles	Fat accumulation occurring following RC tear (different process than metabolic lipid storage: ectopic fat accumulation that occurs in chronic RC tears does not occur by activation of canonical intramyocellular lipid storage and synthesis pathways)	Aging-associated exacerbation in fatty degeneration and infiltration following rotator cuff tear
				Rotator cuff tear reduces muscle fiber specific force production and induces macrophage accumulation and autophagy
miR-133a miR-499	Promotion of myogenesis, myoblast differentiation and muscle adaptation	Downregulated in torn RC muscles	Muscle atrophy and reduction in muscle fiber force production	Aging-associated exacerbation in fatty degeneration and infiltration following rotator cuff tear
miR-138	Suppression of adipogenesis process by inhibiting the translation of C/EBPa mRNA in adipocytes	Upregulated in torn RC muscles	Fat accumulation occurring following RC tear (different process than metabolic lipid storage)	Rotator cuff tear reduces muscle fiber specific force production and induces macrophage accumulation and autophagy
miR-140-3p	Negative regulation of nuclear receptor coactivator 1 (NCOA1) and nuclear receptor-interacting protein 1 (NR1P1) expression, which are coactivators of NF-kB and its inflammatory pathway	Downregulated in degenerative RC tear and in RC chronic tendinopathy (progressive decline in the expression levels depending on the severity of tendon degeneration)	Tendon inflammation and degeneration	MicroRNA profiling reveals distinct signature in degenerative rotator cuff pathologies
miR-140-5p	Downregulation of VEGFA levels	Downregulated in RC tear	Increased VEGFA levels which promote tenogenic differentiation of TDSCs, collagen formation, angiogenesis, EMC formation and tendon-bone healing	Long noncoding RNA H19 accelerates tenogenic differentiation by modulating miR-140-5p/VEGFA signaling
miR-145-5p	Suppression of proinflammatory cytokines IL-beta, TFA-alfa and IL-6 and antiapoptotic role	Downregulated in rc injury with fatty infiltration	Tenocyte apoptosis, ECM disorganization. Fatty infiltration and inflammation in rotator cuff tendon injuries	Genes interconnecting AMPK and TREM-1 and associated microRNAs in rotator cuff tendon injury
miR-192-5p	TGF-beta dependent profibrotic role	Downregulated only in degenerative RC tear and not in rotator cuff chronic tendinopathy	Fibrosis in rotator cuff chronic tendinopathy	MicroRNA profiling reveals distinct signature in degenerative rotator cuff pathologies
miR-205-5p	Downregulation of VEGFA levels	Not reported	Downregulation of tendon-bone healing process, through inhibition of tenocyte proliferation and migration and Col1, Col2, Col 3 expression	High expression of VEGFA in MSCs promotes tendon-bone healing of rotator cuff tear via microRNA-205-5p
	Downregulation of MECP2			Inhibition of miR-205 promotes proliferation, migration and fibrosis of tenocytes through targeting MECP2: implications for rotator cuff injury
miR-210-3p	Specific role not reported	Downregulated in degenerative RC tear and in RC chronic tendinopathy (progressive decline in the expression levels depending on the severity of tendon degeneration)	Specific role not reported	MicroRNA profiling reveals distinct signature in degenerative rotator cuff pathologies
miR-222-3p				
miR-221	Promotion of ECM synthesis	Upregulated in torn RC muscles	Fibrosis	Aging-associated exacerbation in fatty degeneration and infiltration following rotator cuff tear
miR-221	Specific role not reported	Upregulated in torn RC muscles	Fat accumulation occurring following RC tear (different process than metabolic lipid storage)	Rotator cuff tear reduces muscle fiber specific force production and induces macrophage accumulation and autophagy
		Downregulated during adipogenesis in adipocytes		
miR-297	Immunomodulatory function through IL-4 and IL-10 expression and negative control of NF-kB activation	Upregulated in RC injury with fatty infiltration	Immunomodulatory function	Genes interconnecting AMPK and TREM-1 and associated microRNAs in rotator cuff tendon injury
miR-324-3p	Downregulation of metalloproteases expression (eg, MMP-2 and MMP-9)	Downregulated in degenerative RC tear and in RC chronic tendinopathy (progressive decline in the expression levels depending on the severity of tendon degeneration)	ECM degradation and tendon disorganization	MicroRNA profiling reveals distinct signature in degenerative rotator cuff pathologies
miR-425-5p	Negative regulation of inflammatory cytokines (eg, IL-2)	Downregulated in degenerative RC tear and in RC chronic tendinopathy (progressive decline in the expression levels depending on the severity of tendon degeneration)	Tendon inflammation and degeneration	MicroRNA profiling reveals distinct signature in degenerative rotator cuff pathologies

*RC*, rotator cuff; *ECM*, extracellular matrix; *TDSCs*, tendon-derived stem cells; *AMPK*, adenosine monophosphate-activated protein kinase; *TREM*, triggering receptors expressed on myeloid cells; *IL*, interleukin; *MMP*, matrix metalloproteinases; *VEGFA*, vascular endothelial growth factor A; *MECP2*, methyl-CpG binding protein 2; *Col*, collagen type.

Plachel et al^25^ conducted a clinical study examining five patients with degenerative RCT, five patients with chronic rotator cuff tendinopathy, and four healthy controls. They identified several miRNAs significantly deregulated in both chronic tendinopathy and degenerative RCTs: miR-30a-5p, miR-140-3p, miR-210-3p, miR-222-3p, miR-324-3p, and miR-425-5p. Their expression levels showed a progressive decline depending on the severity of tendon degeneration, highlighting their potential role in the pathogenesis and progression of degenerative rotator cuff pathology. They also identified a miRNA signature specific for degenerative rotator cuff pathologies, represented by 6 circulating miRNAs (3%) significantly downregulated only in patients with degenerative RCTs when compared with both healthy controls and patients with chronic rotator cuff tendinopathy (miR-18b, miR-19a, miR19-b, miR-25, miR-93, miR-192).

Liu et al^16^ in their study explored the relationship between tendon-derived stem cells (TDSCs) and vascular endothelial growth factor A (VEGFA) and their epigenetic regulation. TDSCs present spontaneous tenogenic differentiation potentials *in vitro*,^29^ thus indicating reparative properties in tendon injury. Several studies have demonstrated VEGF upregulation in the bone-tendon junction during acute injury healing,^34^ during cruciate ligament reconstruction in dogs, and at the surgical sites of leg bones in rats.^40^ In their study, using *in vitro* cultured TDSCs and *in vivo* rotator cuff tear rat model, they demonstrated a regulatory axis in which the long noncoding RNA H19 targets and suppresses miR-140-5p, thus upregulating the expression of the downstream target VEGFA axis to promote the tenogenic differentiation of TDSCS in RCT and in tendon-bone healing.

Xu et al^37^ in an RCT rat model highlighted the role of microRNA-205-5p in downregulating VEGFA levels and the correlation between microRNA-205-5p knockdown and a substantial increase in VEGFA levels, with the subsequent promotion of the tendon-bone healing process, expression of collagen type I (Col1) and collagen type II (Col2), and elevation of ultimate load of failure and stiffness. Furthermore, a subsequent *in vitro* study by Mao et al^18^ demonstrated the role of miR-205 in the downregulation of methyl-CpG binding protein 2 (MECP2). MECP2 is an epigenetic regulator widely reported to promote tenocyte differentiation, proliferation, migration, and the expression of known markers of fibrosis (Col1, Col3, α-SMA, scleraxis, and tenascin C), which are pivotal for wound healing. Thus, its downregulation by miR205 severely compromises the tendon-bone healing process.

Millar et al^22^ using *in vitro* tenocyte cultures and *in vivo* models of tendon damage demonstrated that miRNA-29a’s effect in altering COL1/3 expression is regulated by Interleukin 33 (IL-33) expression. IL-33 is an “alarmin” expressed in early tendinopathy, tendon injury and subsequent tissue remodeling. *In vivo* tendon healing model they demonstrated an increase in IL-33 levels and a corresponding decrease in miR-29a on days 1 and 3, without significant difference by day 7, supporting the concept of IL-33 as an early tissue mediator in tendon injury. On repeated micro-injury, IL-33 is released, resulting in its constant upregulation, miR-29a suppression, and subsequent decrease in tensile strength of tendon tissue and tendon load to failure. Furthermore, they also demonstrated that in an inflammatory environment with high levels of IL-33, simply supplementing tenocytes with miR-29a was sufficient to inhibit the increased production of collagen 3, thus highlighting its role as a key posttranscriptional regulator of collagen synthesis, tendon healing, and tissue remodeling in murine and human tendon injury. Their result is coherent with several prior studies investigating the role of miR-29 family in ECM composition.^2^^,^^3^^,^^7^^,^^26^^,^^28^

Ko et al^12^ in their clinical study examined 57 patients with RC lesions with (n = 22) and without (n = 35) shoulder stiffness. They demonstrated a significant downregulation of miR-29a in the subacromial bursa and in blood samples in the stiffness group (*P* < .05), thus evidencing that miR-29a signaling loss is correlated with subacromial bursitis in the development of rotator cuff tear with shoulder stiffness. They also demonstrated that the administration into the injured site of mice with rotator cuff lesions of Lentivirus human miR-29a precursor significantly attenuated the tenotomy-induced vessel formation, COL3 synthesis, and fibrotic tissue development, confirming its indispensable role in fibrotic matrix overproduction in the inflamed subacromial bursa.

Leal et al^14^ examined miR-29 expression in tendon samples of 40 patients undergoing arthroscopic rotator cuff repair for full-thickness supraspinatus tear. They found that although miR-29a-3p, miR-29b-3p, and miR-29b-5p were inversely correlated with different MMPs (MMP1, MMP2, MMP9, and MMP14), their expression levels did not differ between injured and noninjured tendons (*P* >.05). They concluded that these miRNAs do not seem to be the main contributing factors regulating the ECM genes studied.

Gumucio et al^10^^,^^11^ measured miRNAs involved in muscle atrophy, lipid accumulation, and matrix synthesis in an in vivo study using a murine model of RCT injury. Thirty days after inducing a tear, they observed a marked increase in lipid accumulation within and around muscle fibers and in fatty macrophage deposition, with a concomitant increase in the expression of peroxisome proliferator-activated receptor gamma (PPARγ) and cytosine-cytosine-adenosine-adenosine-thymidine/enhancer binding protein alfa (C/EBPα), which are key regulators of adipogenesis and have been shown to have a broad overlap in their transcriptional targets.^27^ PPAR-γ also plays an important role in promoting the differentiation of macrophages into foam cells.^33^ Surprisingly, at the same time, they also found upregulated levels of miR-221, which is downregulated during adipogenesis,^35^ and miR-130a and miR-138, known to suppress adipogenesis by inhibiting the PPAR-γ and C/EBPα pathways, respectively,^15^^,^^39^ thus providing further insight into the differences between metabolic lipid storage and fat accumulation following rotator cuff tears.

Thankam et al^31^ examined eight patients with RTCI, comparing four patients with inflammation and fatty infiltration and four patients without. They identified 13 miRNAs (12 downregulated: hsa-miR-145-5p, hsa-miR-99a-5p, hsa-miR-100-5p, hsa-miR-150-5p, hsamiR-193b-3p, hsa-miR-103a-3p, hsa-miR-31-5p, hsa-miR-195-5p, hsa-miR-497-5p, hsamiR-15a-5p, hsa-miR-16-5p, hsa-let-7b-5p, 1 upregulated: hsa-miR-297) involved in the adenosine monophosphate-activated protein kinase (AMPK)-mediated fatty infiltration and Triggering Receptors Expressed on Myeloid cells (TREM)-1-mediated inflammatory response, identifying epigenetic regulators that could potentially be targeted to reduce fat tissue infiltration and inflammation causing muscle-tendon unit stiffness, tendon physiology and mechanics impairment, and healing response compromise.

In another study,^30^ they also identified 10 miRNAs (8 downregulated: hsa-miR-145-5p, hsa-miR-100-5p, hsa-miR-23b-3p, hsa-let-7d-5p, hsa-miR-146a-5p, hsa-miR-150-5p, hsa-miR-181a-5p, and hsa-miR-193b-3p, 2 upregulated: miR-498 and miR-297) associated with the activation of a battery of proinflammatory genes dependent on JAK2/STAT3 pathway in RCTs associated with glenohumeral arthritis, correlating shoulder tendon inflammation with coincidence and severity of glenohumeral arthritis, and suggesting a potential target for future therapies in the management of shoulder pathology.

## Discussion

### Prevalence and risk factors of rotator cuff tendon injuries

Rotator cuff injury is an important disease both from an epidemiological point of view, due to its high prevalence, and from a social and economic point of view with high direct and indirect costs for the most industrialized countries.^21^ It has been shown that rotator cuff injury has a prevalence of 20.7% and represents one of the main causes of pain in orthopedic field.^38^ In the general population, the rotator cuff injury is more frequent in the upper limb most frequently used, in the male sex, in the shoulder engaged in heavy and repeated activities, and in traumatic outcomes. The rotator cuff tear etiology is either of an intrinsic or extrinsic type. The extrinsic factors are subacromial impingement, traction overload, repetitive stress, or trauma. Instead, the intrinsic factors are reduced vascularity, impaired tendon structural development, matrix composition, and aging. Both extrinsic and intrinsic factors act synergistically in the occurrence of rotator cuff tears. However, extrinsic factors are more closely associated with the lesions of younger patients. Rupture of the rotator cuff sometimes occurs with an acute injury or traumatic episode, but most injuries are the result of age-related degenerative changes. In fact, the incidence of rotator cuff injuries is increasing in the elderly population, particularly over 60 years.

### Roles of miRNA in physiopathology of rotator cuff tendon injuries

The pathogenesis analysis of the rotator cuff lesions has become increasingly important. Nowadays, the attention is focused on the biological and molecular mechanisms underlying the degenerative alteration that characterizes this pathology. The evaluation of cellular gene expression has evolved from the macroscopic analysis of tissue degeneration to the evaluation of cellular gene expression as a result of the most advanced methods of cellular biological research. Among the most studied gene targets in rotator cuff pathology are miRNAs, small noncoding RNAs that modulate gene expression by binding the messenger RNA ie, involved in the transcription of the genome. The miRNA seems to be involved in regulating the cellular architecture of the rotator cuff injury. Moreover, it appears to determine several changes in muscle atrophy, adipose involution, and fibrous tendon injury.

### Fat infiltration

Fatty degeneration represents a condition of tissue change ie, found in the rotator cuff that has suffered damage.^9^ Fat infiltration has a direct association with poor outcomes in terms of recovery and surgical healing, but the pathogenetic mechanisms underlying it are poorly understood. Fatty infiltration usually begins in the muscle region of the rotator cuff and often affects the rotator cuff tendons.^32^ Therefore, the accumulation of fat that follows the rupture of the rotator cuff is a biological process different from the metabolic accumulation of intracellular lipids. In fact, the accumulation of ectopic fat in rotator cuff lesions does not occur by activation of the normal pathways of accumulation and synthesis of lipids.

### Inflammatory response, fibrosis, and muscle atrophy

MiRNAs are extremely important in the inflammatory response at the level of the rotator cuff lesion. In particular, it has been shown the role of miRNA in the effects that inflammation has on tissue evolution at the level of the lesion, especially towards fibrosis. The analysis of miRNA subsets proved that the hypoexpression of certain miRNAs is the basis of the phenomena of subversion of the cellular matrix, the increased inflammatory response, and the fibrotic development of the rotator cuff subjected to injury or chronic insult.^25^ In the future, the knowledge acquired could be used as potential diagnostic and prognostic biomarkers for both chronic tendinopathy and degenerative rotator cuff pathologies.

### MicroRNA involved in healing process

In addition to the evaluation of miRNAs role in the pathogenesis of the lesion of the rotator cuff, it has also been considered the role of miRNAs in the positive regulation of the healing processes of the tendon. Liu et al^16^ showed that miR-140-5p has a downregulation function of VEGF levels. This miRNA is hypoexpressed in rotator cuff lesions, resulting in an increase in the levels of VEGF. Therefore, there is an increase in VEGF effect, which promotes tenogenic differentiation of TDSCs, collagen formation, angiogenesis, the creation of ECM, and healing of the tendon-bone.

### Possible future perspectives for clinical use

From a careful analysis of the literature, we discovered that miRNAs studied for the rotator cuff lesion have also been found in other types of lesions, both cancerous and nontumorous, and in the evolutionary processes that characterize them. To date, there are research panels for clinical use for the diagnosis of various tumor pathologies, but a specific research panel has not yet been created in the field of tendinopathies. The clinical use of miRNAs is still initial, and our study aims to catalog the genes and their mediators involved in rotator cuff injury with the intention of guiding biological research in a targeted way for a rapid response both in the diagnostic and therapeutic fields.

## Conclusion

In the last decade, it has been shown that gene expression may be altered in the pathological processes of tendon lesions. The knowledge of all the gene mechanisms underlying rotator cuff tendinopathy should be achieved with future diagnostic and clinical studies. With the advent of modern techniques that enable genome parts to be engineered and used to prevent disease, it becomes essential to know how to treat, prevent, and diagnose more accurately.

## Disclaimers:

Funding: No funding was disclosed by the authors.

Conflicts of interest: The authors, their immediate families, and any research foundation with which they are affiliated have not received any financial payments or other benefits from any commercial entity related to the subject of this article.
